# In vitro gametogenesis in the ongoing quest to vanquish infertility: the role of epigenetics

**DOI:** 10.1016/j.xfre.2024.10.001

**Published:** 2024-10-09

**Authors:** Eli Y. Adashi, Gary M. Wessel

**Affiliations:** a Warren Alpert School of Medicine, Brown University, Providence, Rhode Island; b Division of Biology and Medicine, Department of Molecular and Cellular Biology, Brown University, Providence, Rhode Island

Human conception resulting in a live birth is an inefficient process that has instigated a variety of assisted technologies, and recent experimental work in the mouse model appears to open up new possibilities for the future treatment of human infertility. Investigators are now capable of developing eggs and sperm in vitro, so-called in vitro gametogenesis (IVG) (1), derived from induced pluripotent stem cells (iPSCs). Each step of the IVG process in the mouse iPSCs is selected for; the resulting oocytes display high fertilizability, and the embryos are capable of implantation and result in live births. The technology of IVG forces us to reimagine what may be possible in the fertility field and includes same-sex conception (2), expanded fertility in patients with abnormal ovarian development, and even obtaining eggs from fibroblasts of postmenopausal women. As remarkable as this technology is, its efficiency remains unacceptably low; only approximately 4% of transferred embryos derived from iPSC gamete procedures result in a live birth. Why are these rates so low compared with those in traditional in vitro fertilization in the mouse? What are we missing in the state-of-the-art technologies and successes?

Epigenetics is known to be crucial in the development and maintenance of many different cell types, embryo stages, and developmental processes. Epigenetics refers to the modifications of the DNA, and the proteins involved in wrapping DNA into stable structures of nucleosomes and even higher-ordered chromatin structures. Epigenetic modifications include DNA methylation of cytosine and guanine residues, usually in the promoter region of a gene, and, thereby, turning off the transcription of that gene. DNA methyltransferases are endogenous enzymes of cells that are directed to carry out this type of epigenetic modification. So too the histones that DNA wrap around for packaging can be modified in diverse biochemistries: phosphorylation; methylation; acetylation; and many other covalent modifications. As a consequence of sometimes opposing activities, these modifications will increase or decrease transcription of the gene where these modifications are installed. Eggs and sperm possess epigenetic markers from their developmental program during gametogenesis, and these “leftover” signals are usually wiped clean during early development, only to be replaced by a different set of epigenetic markers. In the absence of these alterations, the early embryonic cells receive mixed messages as well as incomplete instructions as to how to successfully live up to their task with high fidelity as a gamete and early embryo.

Although IVG in the mouse can result in large numbers of eggs, those eggs and the resultant embryonic cells often do not replicate the dynamic changes in epigenetic signatures normally seen in the authentic cells. Research suggests that the low reproductive efficiency of IVG in the mouse iPSCs is a result of epigenetic malfeasance and is a major barrier to success of human iPSC-derived primordial germ cells (PGCs). However, thus far, no reports of successful human IVG have been made.

In their most recent work, Murase et al. (3) analyzed the very low success rate that they and others have reported to convert human iPSC into PGC-like cells. One possible explanation for this result is that these cells seldom attain a semblance of the epigenetic markers of authentic PGCs and the induced PGC-like cells never fully acquire a germline assignment. Why?

They approached an explanation from the perspective of signaling molecules and how important cell-cell communication is during development, cell migration, and epigenetic reprogramming. They screened conditions that are thought to be involved in the epigenetic state of authentic primordial germline cells, and remarkably, when the PGC-like cells are cultured with the signaling factor bone morphogenic protein-2 (BMP-2), several authentic epigenetic transitions occurred. Bone morphogenic protein-2 is a member of the family of transforming growth factor, transforming growth factor-β, signaling molecules that often result in changes in transcriptional activity in the receiving cells. The BMP-2 signaling cascade resulted in the expression of several genes, the enzymatic activities of which modify the epigenome of PGC-like cells, thereby enabling their progress as germline cells. The researchers incorporated single cell RNA-sequencing approaches and sequencing for DNA methylation patterns in the PGC-like cells.

Of great interest here for considering the normal conditions of germline formation was that BMP-2 proved effective only at its medium test dosage—more or less of this factor minimized the reprograming success. Such concentration-dependent responses to a signaling factor means that a single signaling factor can impact acquisition of different cell fates; PGC-like cells may form with the optimal dose, whereas perhaps a variety of somatic cell fates may develop at different doses. Furthermore, multiple metrics for successful PGC-like development included expression of genes that only occur on epigenetic reprogramming and germline progression into the stem populations. These key gene expression patterns, which are dependent on the epigenetic reprograming, include expression of the RNA binding protein DAZL (deleted in azoospermia-like) and the RNA helicase DDX4 (Vasa), both of which are associated with germline formation in many animals. However, most importantly, BMP-2 treatment enabled PGC-like cells to expand greatly, acquire more characteristics of native PGCs, and develop into stem populations of oogonia and spermatogonia. The human PGC-like cells did not reach the next developmental step of authentic germ line stem cells; however, they did transition further in this progression in vitro than ever seen before for human PGC-like cells.

The advancements described herein raise hope through the community that IVG is closer to human application than we thought. Being able to modulate epigenetic reprogramming increases the potential that future eggs and sperm derived from these in vitro approaches will be of high fidelity such that at some point, IVG may become a possibility in humans. This study also points out the limited utility that mice have constituting a model of the germ line. Many of the epigenetic changes and mechanisms leading to the epigenome are different between mice and humans, thereby rendering this study of human-derived iPSCs essential to the research progress in this field. It is not that human IVG is more difficult scientifically, but the ethical issues of replicating a systematic plan to test IVG in humans, as was accomplished in mice, dominate such a plan. If the goal is to have IVG a future clinical option for infertility, it will be essential to continue to advance this research effort with human cells so as to compel progress into a clinical reality.

## CRediT Authorship Contribution Statement

**Eli Y. Adashi:** Writing – review & editing, Writing – original draft, Conceptualization. **Gary M. Wessel:** Writing – review & editing, Writing – original draft.

## Declaration of Interests

E.Y.A. has nothing to disclose. G.M.W. has nothing to disclose.
